# Excretion of Heavy Metals and Glyphosate in Urine and Hair Before and After Long-Term Fasting in Humans

**DOI:** 10.3389/fnut.2021.708069

**Published:** 2021-09-28

**Authors:** Franziska Grundler, Gilles-Eric Séralini, Robin Mesnage, Vincent Peynet, Françoise Wilhelmi de Toledo

**Affiliations:** ^1^Buchinger Wilhelmi Clinic, Überlingen, Germany; ^2^Charité-Universitätsmedizin Berlin, Corporate Member of Freie Universität Berlin, Humboldt-Universität zu Berlin and Berlin Institute of Health, Berlin, Germany; ^3^Department of Biology and Network on Risks, Quality and Sustainable Environment MRSH, University of Caen Normandy, Caen, France; ^4^Gene Expression and Therapy Group, Department of Medical and Molecular Genetics, King's College London, Faculty of Life Sciences and Medicine, Tower Wing, Guy's Hospital, London, United Kingdom; ^5^Institut de Recherche et d'Expertise Scientifique, Europarc, Strasbourg, France

**Keywords:** Buchinger Wilhelmi fasting, weight loss, arsenic, nickel, lead, glyphosate, biomonitoring

## Abstract

**Background:** Dietary exposure to environmental pollutants in humans is an important public health concern. While long-term fasting interrupts the dietary exposure to these substances, fat mobilization as an energy source may also release bioaccumulated substances. This was, to our knowledge, only investigated in obese people decades ago. This study explored the effects of 10-days fasting on the excretion of heavy metals and glyphosate.

**Methods:** Urinary levels of arsenic, chromium, cobalt, lead, nickel, mercury and glyphosate were measured before and after 10 fasting days in 109 healthy subjects. Additionally, hair analysis was done before and ten weeks after fasting in 22 subjects.

**Results:** Fasting caused a decrease in body weight, and in urinary arsenic (by 72%) and nickel (by 15%) concentrations. A decrease in lead hair concentrations (by 30%) was documented. Urinary mercury levels were unchanged for chromium, cobalt and glyphosate, which were undetectable in most of the subjects. Additionally, fatigue, sleep disorders, headache and hunger were reduced. Body discomfort symptoms diminished four weeks after food reintroduction.

**Conclusions:** The results of this study provide the first insights into the changes in heavy metal excretion caused by long-term fasting. Further studies focusing on the kinetics of efflux between different compartments of the body are needed.

**Clinical Trial Registration:** https://www.drks.de/drks_web/navigate.do?navigationId=trial.HTML&TRIAL_ID=DRKS00016657, identifier: DRKS00016657.

## Introduction

The exposure to heavy metals and pesticides in human populations has increased over the last decades. The main source of contamination by these substances is environmental pollution caused by anthropogenic activities. This includes industries like petroleum refineries, mining, electroplating, painting, or the production and use of synthetic chemicals like pesticides and fertilizers (1). When they are lipophilic, environmental pollutants can also bioaccumulate along the food chain making the diet a significant contributor to human exposure (2).

Heavy metals are defined either according to their high atomic weight or to their high density (above 5 g/cm^3^). They are naturally deposited in the ground water and soil (3), and also enter the life cycle and organisms through contaminated food intake (2). Small quantities of metals such as zinc, nickel or cobalt are essential for physiological functions in the human body, e.g., as co-factors of key enzymes or oxidation-reduction reactions (4). However, exposure to higher concentrations through oral ingestion, dermal contact or inhalation can be toxic for human health (3). Some heavy metals accumulate in the body and are stored in various tissues, including the adipose tissue (5). They can also be found in hair (6, 7). Heavy metals are non-biodegradable (3). Toxic levels of arsenic, cadmium, chromium, cobalt, lead, nickel and mercury can trigger DNA damages and structural changes of cellular components either directly through interacting with the organic molecules, or indirectly through production of reactive oxygen species (ROS), leading to various diseases such as cancer, neurological abnormalities, cardiovascular diseases, hormonal diseases and infertility (3). For instance, a recent study showed that urinary arsenic and cadmium associate with vascular brain injury (8). The toxicity mechanisms common to metals and determining their toxicity is the generation of oxidative stress by the production of reactive oxygen and nitrogen species (9).

The exposure to some pesticides has been shown to cause adverse health effects in human populations after acute intoxications (10), repeated occupational exposures (11), or environmental exposures during sensitive periods of the development like pregnancy (12). The toxicity of some pesticides can be amplified by the presence in formulations of other compounds with hazardous properties, as in the case of glyphosate (13, 14). Recent studies also reported that some glyphosate formulations are contaminated by polycyclic aromatic hydrocarbons and heavy metals (1, 15). We focused on glyphosate which is the main declared ingredient used in pesticide formulations worldwide (16). It is generally admitted that approximately 20% of the absorbed glyphosate is excreted in urine after exposure to high concentrations in animal studies. However, recent studies bring contradictory results concerning dietary exposure in humans. It is thus still unclear how the urinary excretion of glyphosate reflects the daily intake (17–19).

The use of dietary supplements in so-called “detox diets” is frequently advocated to mitigate the adverse effects of toxic exposures. Molecular mechanisms of xenobiotics detoxification are well-characterized (20). The detoxification of xenobiotics is a multistep process occurring in different tissues, predominantly in the liver. The different steps consist in a succession of enzymatic reactions which activate (mostly by redox reactions), conjugate and excrete xenobiotics. While some evidence is available to support the detoxifying properties of some plants in laboratory animals (21), limited evidence supports the claim for beneficial effects of detox diets because they are generally not tested with properly designed clinical trials in humans (22). Only one study describes toxic trace element detoxification caused by a switch to an organic plant based diet in a multi-armed randomized clinical trial (23). In addition, some herbal remedies produced with poor-manufacturing practices can also cause liver injuries (24) because they are frequently contaminated with heavy metals (25). The most effective approach to mitigate toxic effects of environmental pollutants is to provide a diet certified free of pollutants. A switch to an organic diet is also sometimes advocated to have health benefits because the consumption of fruits and vegetables with high levels of pesticide residues has been linked to various adverse health outcomes or poor semen quality (26).

Fasting treatments are often associated in the public for their detoxifying properties but only few studies document it. Some of them observed long-term fasting, defined as voluntary interruption of food intake for at least 2 days up to several weeks (27). A general improvement of health in subjects following this fasting program was found (28–31). Moreover, an increase in antioxidant capacity and reduced lipid peroxidation was documented in the cohort we describe in this article (32, 33). However, it was still unclear whether fasting has a measurable detoxifying effect. In this study, we analyzed the presence of heavy metals and glyphosate in urine before and after a 10-days fasting period. Additionally, since metal concentration in hair correlate well with blood levels they are thus known to be a reliable biomonitoring strategy (34). We proceeded to an exploratory hair analysis in a subgroup of subjects.

## Materials and Methods

### Ethics Statement

This prospective, observational study was conducted in accordance with the Declaration of Helsinki. The medical council of Baden-Württemberg, Stuttgart, approved the study protocol on 12 February 2019 (application number F-2018-118) and it was registered on 20 February 2019 in the German Clinical Trials Register (DRKS-ID: DRKS00016657). Subjects were recruited between 15 September 2019 and 18 November 2019. All participants gave their written informed consent before enrolling into the study. After the 10-days fasting period at the clinic, an online follow-up took place 4 weeks after the fasting treatment.

### Participants

Participants had to fulfill the following inclusion criteria as described previously (32, 33) in order to be enrolled: subjects had to be between 18 and 70 years old, and underwent a fasting treatment of 10 ± 3 days. A 10-days fasting period showed in previous studies beneficial health effects (28, 29). A laboratory analysis including blood and urine sampling was done at the start, and a second sampling at the end of the fasting. The intake of micronutrient supplements was advised to be stopped already 1 week before and during the fast. An exception was made for the mineral magnesium in the form of magnesium citrate (29%) and magnesium oxide (26%), because the clinical routine has shown that the supplementation during fasting minors the risk of muscle cramps that could be provoked by high amounts of liquid intake (3 L per day, see protocol) during fasting. Medical contraindications for fasting led to exclusion as described in the guidelines of fasting therapy (35). Participants had to speak German, English or French, to understand the questionnaires, and not to participate in another study.

### The Fasting Protocol

The fasting protocol was precisely and previously described (29). It was conducted under medical supervision and according to peer-reviewed guidelines (35). One-day prior starting the fast, a transition day with a simplified 600 kcal organic diet was completed. The initiation of the fasting period started with the administration of a laxative (20–40 g Na2SO4 in 500 ml water). During fasting subjects received daily 250 ml organic juice at midday, 20 g honey, and 250 ml vegetable soup in the evening, leading to a daily calorie intake of ~250 kcal. It was recommended to drink at least 2–3 L of water or non-caloric, organic herbal teas. The reintroduction of food occurred stepwise from 800 to 1,600 kcal/day with a vegetarian organic diet. Pesticides were not used in the clinic. The fasting program also included physical exercise and individual physiotherapy (29).

### Clinical Data

Before participating in the fasting program, the subjects were thoroughly examined by a medical doctor. Two examinations were performed in the morning during the fasted state. The baseline examination took place before initiating the fasting period, and the second examination was conducted at the end. All clinical data were captured according to the BWC standards. The body height was assessed with seca 285 (Seca, Hamburg, Germany) and the waist circumference was measured with a measuring tape, placed halfway between the lowest rib and the iliac crest. Body weight was measured (with Seca 704/635, Seca, Hamburg, Germany) by trained nurses, while subjects wore light clothing. The medical team documented possible adverse events in a report form.

### Blood, Urine and Hair Collections and Analysis

Blood and urine samples were collected in the first morning after arrival, and at the 10 ± 3 fasting day. Routine laboratory blood parameters were measured in the laboratory MVZ Labor Ravensburg as previously described (32, 33). Urine samples were collected from the first urine in the morning. The quantification of the heavy metals arsenic, chromium, cobalt, lead, nickel, and mercury in urine, as well as glyphosate, was done in the Medical laboratory Bremen (Bremen, Germany). Heavy metals were quantified with inductively coupled plasma mass spectrometry (ICP-MS) with a detection limit of 0.1 μg/l as previously described (36). Glyphosate was quantified with gas chromatography (GC) with tandem mass spectrometry (GC-MS-MS) to reach levels of detection of 0.1 μg/l as described in (37). In brief, glyphosate was derivatized with a mixture of trifluoroacetic anhydride and trifluoroethanol before separation using a Agilent Technologies GC system 7890. Quantification was then performed with an Agilent 7000 mass spectrometer (MS-MS) operated in negative ion mode.

Hair samples were collected from a subgroup of subjects who did not have colored hair at the beginning of the fast, and 10 weeks afterwards. The second time point was defined after 10 weeks taking into account the growth rate of hair (1 cm/month), the needed time to grow from the follicle to the scalp (2 weeks) and the approx. required 2 cm hair samples for analysis. For sample collection either a strand of hair was cut as close to the scalp as possible, different spots from the head were possible, or body hair (armpits or pubic hair) was collected. The baseline samples with head hair were collected by trained study staff. Body hair samples as well as the second sampling was done by the participants themselves, using a special collection kit provided by the laboratory. The collected hair samples lengthways into the aluminum foil and the cut ends (ends of the hair nearest the scalp) were placed on the narrow part of the foil. Samples were sent to IRES laboratory (Strasbourg, France) for heavy metal analysis.

Hair samples were cut to keep 3–4 cm proximal segment (closest to the scalp). Hair strands were accurately weighted (ca. 100 mg) in polypropylene tube. Hair were then digested with 70% nitric acid (HNO3 trace metal analysis grade) under oxidative condition with hydrogen peroxide (H2O2, 30%). Digestion of hair was performed in ultrasonic bath for 2 h at 60°C and overnight incubation at 60°C. Extracts were diluted with ultrapure water (Millipore) and analyzed by ICP-MS with a PlasmaQuant MS from Analytik Jena for quantification of arsenic, chromium, Cobalt, lead, nickel. Limits of detection (LOD) in water ranged from 0.21 to 0.53 μg/l, while the limits of quantifications (LOQ) ranged from 0.95 to 2.51 μg/l. Uncertainty calculated within two standard deviations (k2) for these measures was 9.6%. Quantification of mercury was done by Atomic Absorption Spectrometry (AAS) with a RA-4300 Mercury Analyser from Envirosciences GmbH. In water, LOD was 0.026 μg/l, and LOQ was 0.126 μg/l. Uncertainty calculated within two standard deviations (k2) for mercury was 15.8%. These corresponded to LOQ in hair of 0.15 ng/mg for arsenic, chromium, cobalt, nickel, lead and 0.015 ng/mg for mercury.

### Self-Reported Data

Self-reported data were captured during the stay in a diary to record the symptoms during the fasting period and an additional questionnaire complemented the life style before and 4 weeks after to approach the long-term effects after the fasting period. At baseline, and 4 weeks after fasting, the subjects completed the medical questionnaire (38) to assess their symptoms experienced in the last 4 weeks. All items were rated from 0 (never or almost never have the symptom) to 4 (frequently have it, effect is severe). The overall sum of each item allowed a ranking into three scores: low symptoms (0–14), moderate symptoms (15–49) and high symptoms (>50). The evolution of the intensity for six frequently mentioned symptoms during long-term fasting (29): fatigue, headache, sleep disorders, hunger, back pain and nausea, was documented daily on numeric self-rating scales from 0 (none) to 10 (very much).

Subjects indicated before and 4 weeks after fasting if they consumed in the last 2 weeks organic food by choosing one of the following categories: no, little, quite often and almost always. The smoking habits were indicated by the subjects before and after fasting and after 4 weeks.

### Statistical Analysis

All the statistics were performed with R version 4.0.0. We treated missing values (below threshold of detection) as recommended by the European Human Biomonitoring Initiative (HMB4EU), described in Harel et al. (39). When the proportion of missing values was below 20%, statistical significances were evaluated with the package lmerTest, with linear-mixed models using sex and BMI as a covariate, and the individual identifiers of the subjects as a random effect. For the metabolites for which more than 80% of observations were below the LOQ, we used a mixed effects logistic regression model (R package lme4) with the samples dichotomized as detected/undetected. *P*-values were not calculated when the number of subjects with values above LOQ were below 20%. Summary statistics values were estimated using the maximum likelihood inference for left-censored values when the proportion of missing values was below 80%. The maximum likelihood was estimated using the function cenmle() from R package NADA. When the proportion of samples with missing values were over 80%, the proportion of samples over the limits of detections were indicated. Differences at baseline between the whole cohort and the hair analysis subgroup were evaluated using a *t*-test for continuous variables or a chi-square goodness of fit test for categorical variables. Correlations were calculated using Spearman's rank-order correlation in R.

## Results

**Figure 1:**
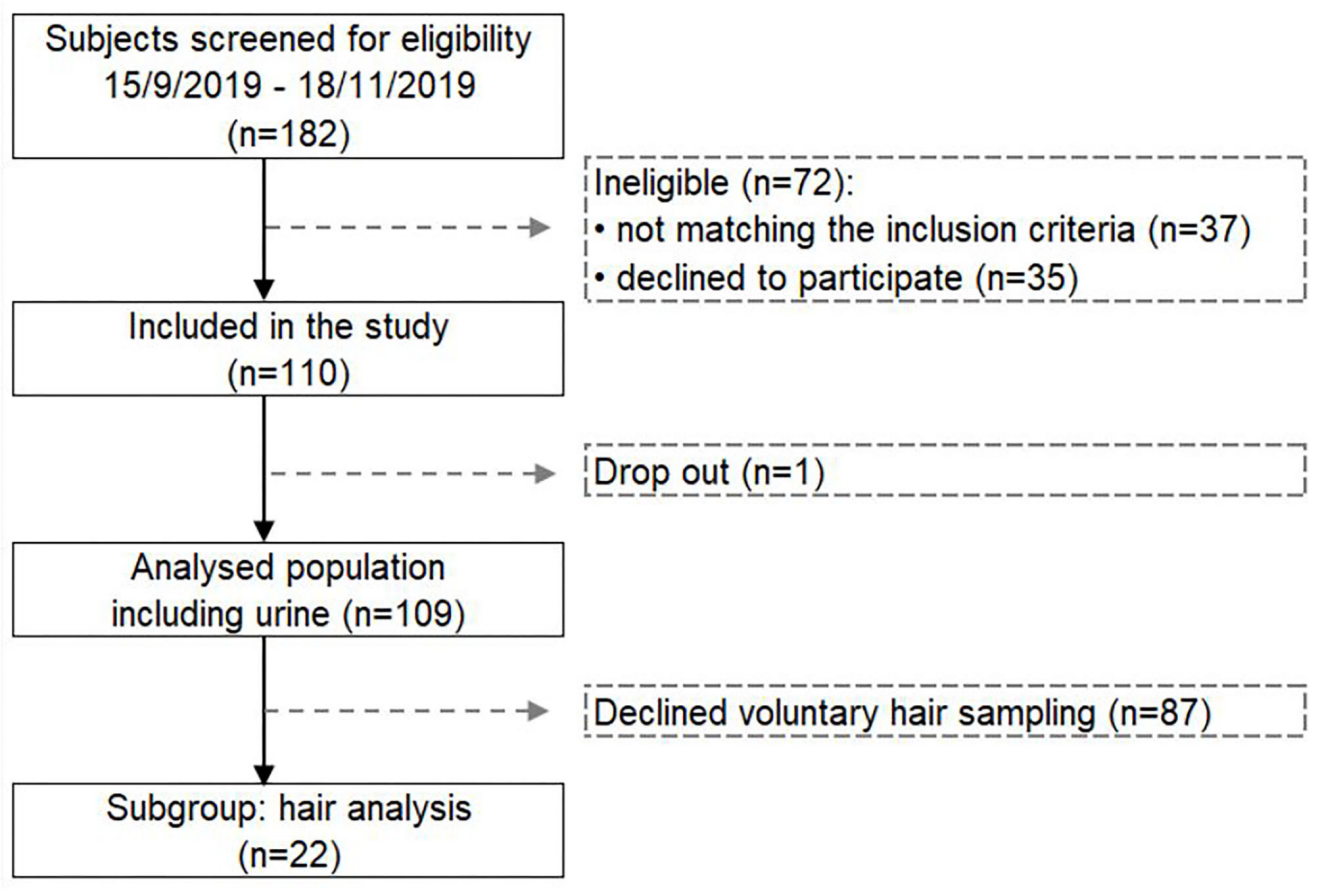
Flow chart presenting the selected study population for measuring the changes in heavy metals and glyphosate excretion during long-term fasting.

Out of the 182 screened subjects, 35 subjects declined to participate and 37 subjects did not meet the inclusion criteria, as described previously (32, 33). In total, 41 men and 68 women with an average age of 56.7 ± 10.4 years participated in the study (Figure 1). The study population was metabolically healthy before, during and after this long-term fasting study. Only one person had to stop the fasting due to low hemoglobin and sodium levels.

The 10-days fasting period led to a mean weight loss of 4.9 ± 1.9 kg (*p* < 0.001; Table 1). Four weeks after fasting the weight loss was 6.7 ± 11.9 kg (*p* < 0.001). At baseline, 28.4% of the subjects almost always consumed organic food, 35.8% quite often, and 25.7% declared they eat little organic food. 8.3% mentioned not to eat organic products. Four weeks after fasting the number of subjects eating organic food had increased significantly (*p* = 0.047). In total, 40.4% almost always consumed organic food, 33.9% quite often, and 11.9% little. Only 6.4% indicated not to eat organic food (Table 1). Nineteen subjects (17.4%) indicated to smoke at baseline (Table 1). Out of them only 2 (0.02%) smoked at the end of fasting and 7 after 3 months (0.06%). These results suggest that after voluntary long-term fasting according to this program health consciousness in general has improved.

**Table 1:** Baseline characteristics of the study population as well as weight changes, organic food consumption and smoking habits after fasting.

**Fasting effects**	**Before**	**After 10 days**	**After 4 weeks**
BMI, kg/m^2^	28.3 ± 6.0	27.7 ± 5.2^***^	26.9 ± 5.7^***^
Weight, kg	82.9 ± 18.8	79.9 ± 16.1^***^	74.4 ± 14.2^***^
Estimated organic food consumption, *n* (*p* = 0.047)			
Almost always	31	Organic	44
Quite often	39	during	37
Little	28	food	13
No	9	reintroduction	7
Smoking, n	19	2	7

After 10 days the subjects stopped fasting, underwent in average a 3-days controlled organic food reintroduction period, and left the clinic. P-values are p < 0.001^***^.

### Heavy Metals and Glyphosate in Urine

Heavy metals and glyphosate excretion was measured in urine before and after long-term fasting in each subject.

At baseline, out of six heavy metals measured, only arsenic was detected in all 109 subjects (Table 2). After fasting it was detectable in 104 subjects and the concentration of urinary arsenic levels significantly decreased by 71.5% (*p* < 0.0001). Nickel was present in nearly half of the cohort at baseline and in 28 subjects afterwards. Urinary nickel levels decreased also significantly by 14.6% (*p* = 0.004). In one-third of the cohort (*n* = 40) mercury was over the threshold at baseline, and in 29 subjects after fasting, but the urinary levels remained comparable after fasting (4.3%). Lead was found in one-quarter of the cohort (*n* = 26), and was in only 13 subjects detectable afterwards. The urinary lead levels decreased and were not detectable after fasting. Cobalt and chromium were detected in only a few subjects before and after fasting with urinary levels below quantification.

**Table 2:** Environmental pollutants before and after long-term fasting in urine.

**All subjects = 109**	**Before**			**After**			**Change**
**Metabolite, LOQ**	***n***	**Mean ± SD**	**Max**	***n***	**Mean ± SD**	**Max**	***p*** **-value**
Arsenic, ≥1 μg/l	109	28.7 ± 47.5	354.1	104	7.2 ± 8.4	180.7	^***^
Chromium, ≥1 μg/l	2	ND	7.9	3	ND	13.8	ND
Cobalt, ≥1 μg/l	5	ND	51.7	6	ND	71.7	ND
Lead, ≥1 μg/l	26	1.6 ± 0.4	2.4	13	ND	1.8	ND
Mercury, ≥1 μg/l	40	2.3 ± 1.2	6.2	29	2.4 ± 1.1	7.1	NS
Nickel, ≥1 μg/l	50	2.1 ± 1.0	6.9	28	1.8 ± 0.8	3.8	^**^
Glyphosate, ≥0.1 μg/l	9	ND	0.9	0	ND	ND	ND

The heavy metals measured are presented in alphabetical order in μg/l. Glyphosate, as a marker of the main pesticide of the world, is also indicated in μg/l. The total number of subjects is 109; the number of subjects (n) above the level of quantification is given for each parameter. ND, not determined; NS, not significant. P-values < 0.01 are indicated as ^**^;

p < 0.001 as ^***^.

**Figure 2:**
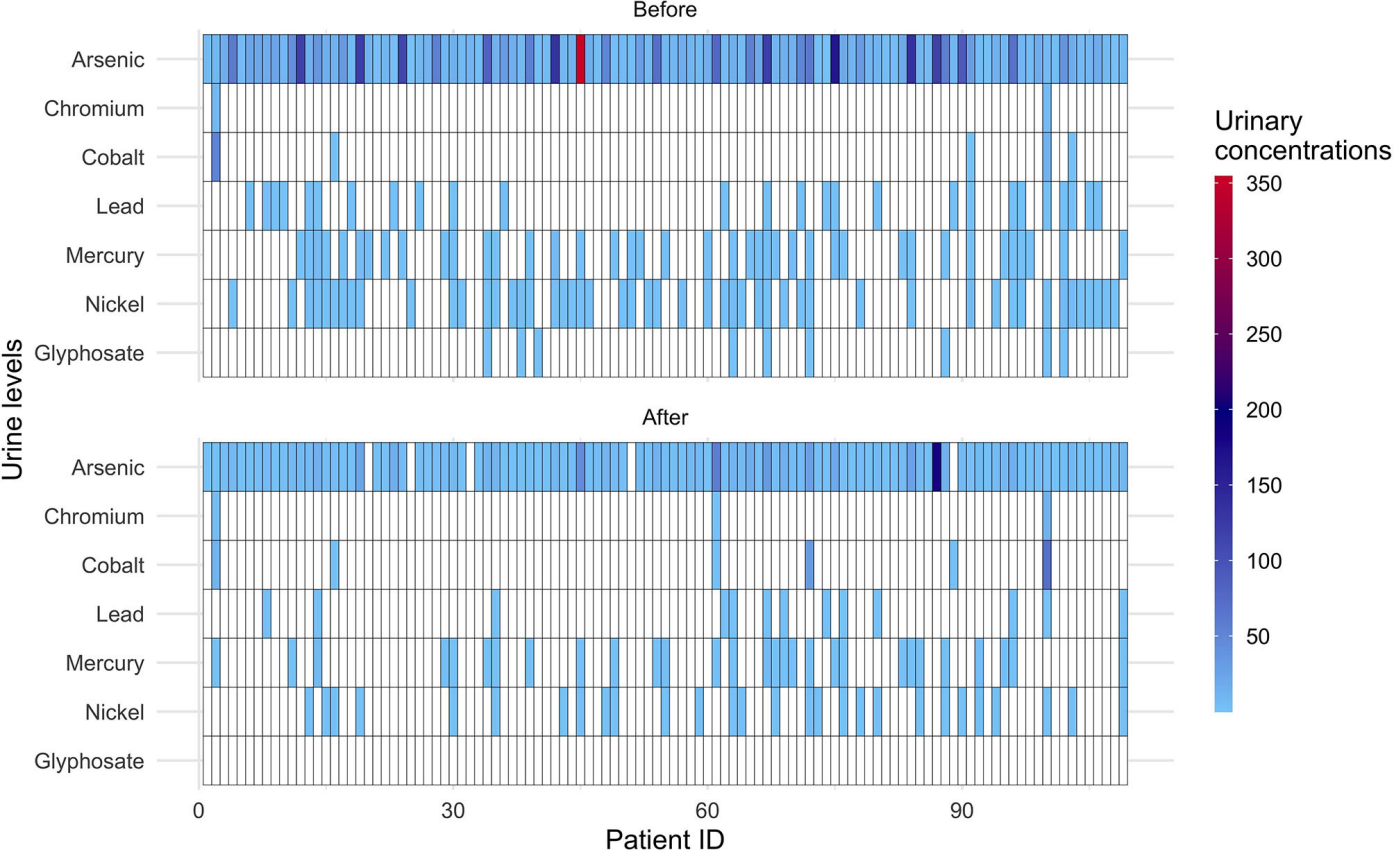
Heatmap displaying the individual excretion of heavy metals and glyphosate before and after 10-days fasting. The color scale shows the urinary levels (μg/l) of heavy metals and glyphosate. Data below detectable levels are indicated in white. Patient ID: individual identification numbers (in total 109).

Nine subjects excreted glyphosate before fasting with levels below quantification. After fasting glyphosate was undetectable in all subjects. A global decrease for most measured compounds in urine is visible in the heatmap (Figure 2).

We also examined correlations between heavy metal concentrations in urine and the BMI as previous studies found that environmental pollutant concentrations in serum are proportional to the weight loss. Arsenic levels were correlated to the BMI of the patients (rho = 0.20, *p* = 0.0045). However, the changes in arsenic levels were not correlated to the weight loss (rho = 0.16, *p* = 0.11). This suggested that the excreted arsenic was stored in fat tissue, and that individuals with a high proportion of body fat have higher impregnation by arsenic, but also that the intensity of the weight loss might have limited relationship with the elimination of arsenic. No relationship between weight loss or BMI were found with the other heavy metals measured.

### Heavy Metals in Hair

Out of the whole cohort, 22 subjects accepted to participate in an exploratory hair analysis. Their mean age of 52.1 ± 12.3 years was lower than that of the whole cohort (*p* = 0.023), less women participated (40.9%; *p* = 0.015) and the subjects had a lower baseline BMI (25.8 ± 4.1 kg/m^2^; *p* = 0.026) compared with the whole cohort.

Hair samples were collected at the beginning and 10.0 ± 2.7 weeks after fasting in order to wait for the hair growth. Arsenic was not detectable in all hair samples before and after fasting (Table 3). Nickel was present in 2 subjects at baseline and in 5 subjects after fasting. At baseline, nickel concentrations in hair were below the quantification level but reached a level of 0.51 ± 0.30 ng/mg 10 weeks after fasting. Mercury could be quantified in all 22 subjects before and after fasting with stable concentrations (2.5%). Before fasting lead was detectable in all subjects except one and in 20 subjects after fasting. The lead concentration in hair decreased significantly by 29.6% (*p* = 0.036). Cobalt was only detected in one subject before and two subjects after fasting with concentrations below quantification levels. Chromium was detected in 19 out of the 22 subjects before and after fasting. The chromium concentration remained comparable (11.4%).

**Table 3:** Environmental pollutants in hair before the 10-days fasting period and 10 weeks afterwards.

**All subjects = 22**	**Before**			**After 10 weeks**			**Change**
**Metabolite, LOQ**	***n***	**Mean ± SD**	**Max**	***n***	**Mean ± SD**	**Max**	***p*** **-value**
Arsenic, ≥ 0.15 ng/mg	0	ND	ND	0	ND	ND	ND
Chromium, ≥ 0.15 ng/mg	19	0.35 ± 0.11	0.61	19	0.39 ± 0.12	0.72	NS
Cobalt, ≥ 0.15 ng/mg	1	ND	0.25	2	ND	0.24	ND
Lead, ≥ 0.15 ng/mg	21	1.08 ± 0.98	3.71	20	0.76 ± 0.62	2.60	^*^
Mercury, ≥ 0.015 ng/mg	22	0.79 ± 0.70	2.90	22	0.81 ± 0.93	3.99	NS
Nickel, ≥ 0.15 ng/mg	2	ND	0.76	5	0.51 ± 0.30	0.97	ND

The heavy metals measured are presented in alphabetical order in ng/mg. On 22 subjects the number of them (n) above the LOQ is given for each parameter. ND, not determined; NS, not significant. P-values < 0.05 are indicated as ^*^.

### Self-Reported Symptoms

**Figure 3:**
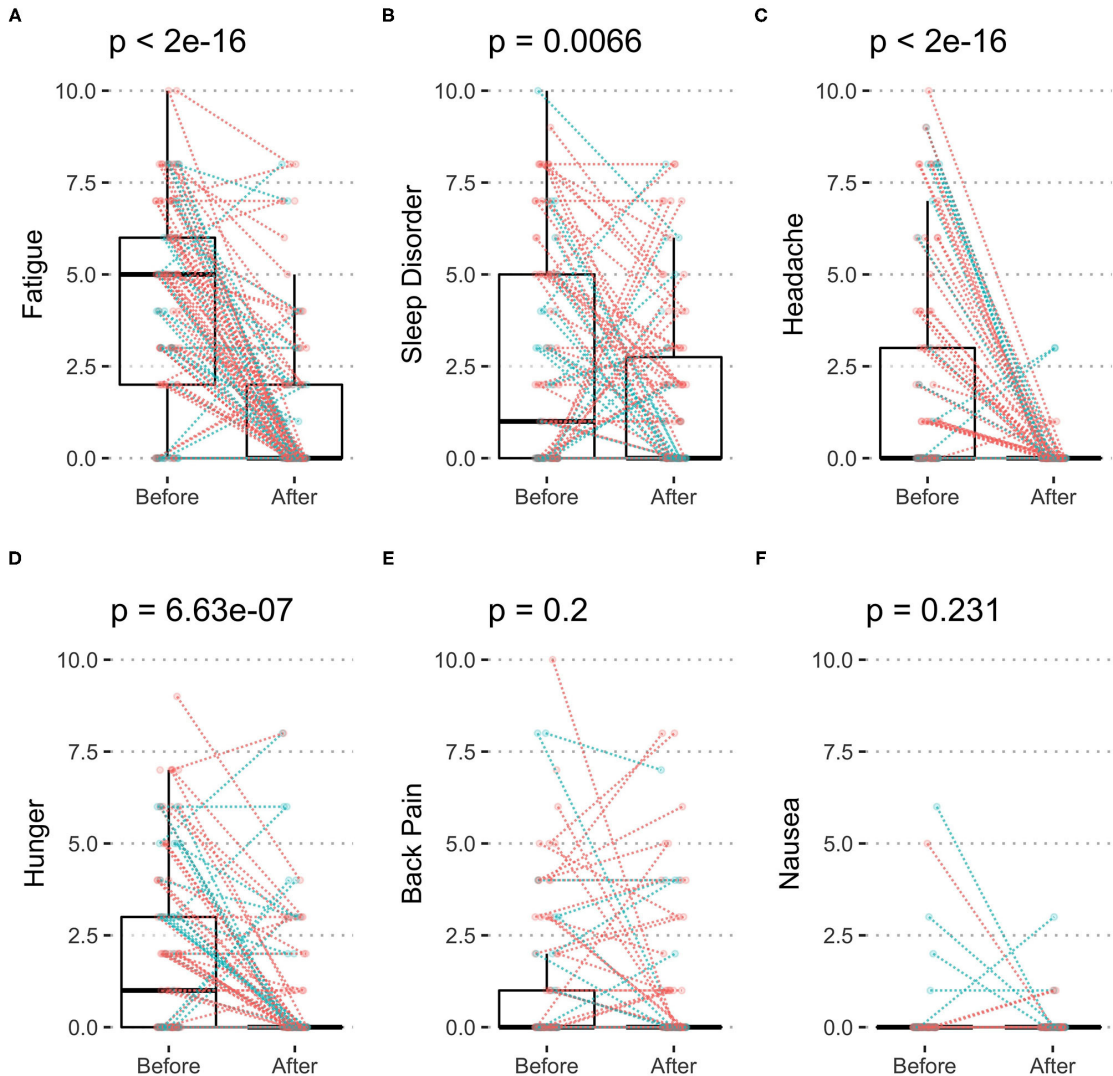
Symptoms of body discomfort before and after 10-days fasting. The individual changes (women in red and men in blue) as well as box plots indicating median and quartiles are presented for fatigue **(A)**, sleep disorder **(B)**, headache **(C)**, hunger **(D)**, back pain **(E)**, and nausea **(F)**.

The most frequent self-reported symptoms at baseline was fatigue with 4.1 ± 2.8 points (Figure 3A; Table 4), followed by sleep disorder with 2.5 ± 1.6 points (Figure 3B) and headache with 2.0 ± 2.9 points (Figure 3C) as well as hunger with 2.0 ± 2.4 points (Figure 3D). Fasting decreased all of them significantly: fatigue by 68.4%, sleep disorder by 35.6%, headache by 96.1%, and hunger by 60.6%. Back pain and nausea (Figures 3E,F) were less frequent and unchanged during fasting.

**Table 4:** Self-reported symptoms before and after 10 fasting days and self-estimated chronic body discomfort before and after 4 weeks of food reintroduction.

**Fasting effects**	**Before**	**After 10 days**	**After 4 weeks**
**Self-reported symptoms, mean ± SD**			
Fatigue	4.1 ± 2.8	1.3 ± 2.1^***^	ND
Sleep Disorder	2.5 ± 2.9	1.6 ± 2.3^**^	ND
Headache	2.0 ± 2.9	0.1 ± 0.4^***^	ND
Hunger	2.0 ± 2.4	0.8 ± 1.7^***^	ND
Back pain	1.0 ± 2.0	0.8 ± 1.8	ND
Nausea	0.2 ± 0.8	0.1 ± 0.3	ND
**Estimated chronic body discomforts**, ***n*** **(*****p*** **= 0.002)**			
Elevated	26	ND	13
Moderate	65	ND	55
Low	15	ND	33

ND, not determined. P-values are <0.01^**^ and

p < 0.001^***^.

The results of the medical symptoms questionnaire indicate, based on 66 items and the specific duration of the symptoms, the symptom profile as high (>50 points) in 23.9%, moderate (15–49 points) in 59.6% and low (<14 points) in 13.8%. Four weeks after fasting high symptoms score were found in only 11.9%, moderate 50.5% and low 30.3%. There was a shift from high and moderate symptoms score to lower score groups, indicating a decrease of these symptoms after fasting (*p* = 0.002; Table 4).

## Discussion

Chronic exposure to environmental pollutants such as heavy metals and pesticides increasingly influences human health (40). We measured how a 10-day fasting period, where virtually no food was absorbed, influenced the excretion of heavy metals and glyphosate. At baseline, heavy metals were detectable in urine, and their concentration reduced by the fasting intervention. Furthermore, the occurrence of symptoms of chronic body discomfort was improved.

Fasting is defined by the temporary cessation of food intake (27). Consequently, the oral ingestion of possibly contaminated food is stopped. The metabolism switches to the usage of fat and ketones, using endogenous energy reserves (27, 41). Urinary levels of heavy metals or pesticides mainly reflect their exposures in the last few days (42). It is known that arsenic and nickel are rapidly absorbed through the gastrointestinal tract (3). Long-term fasting thus interrupts the exposure to dietary pollutants which probably explains at least partly why urinary concentrations of some heavy metals were reduced in our study. Furthermore, the fasting was conducted in BWC, a setting where the exposure to air pollutants from transport or residential exposures to pesticides is very low.

Long-term fasting at BWC can be considered as a health strategy (29) to avoid external contamination with potentially toxic chemicals, because it interrupts the dietary intake of environmental pollutants like heavy metals or glyphosate in this study. However, whether it detoxifies the body in the sense that it decreases pollutant body burden cannot be determined in this study. Some of the heavy metals investigated in this study could have been bioaccumulated from past exposures (2). A recent study demonstrated the presence of nickel, lead, tin, titanium, in 228 adipose tissue samples of humans (43). Mercury was found in half of these same samples. Long-term fasting leads to a significant weight and fat loss, and a reduction in waist circumference, reflecting a decrease in visceral fat tissue (29, 44). It could be hypothesized that pollutants that were accumulated in the fat tissue are slowly released during long-term fasting. In another study, calorie restriction-induced weight loss provoked a release of persistent organic pollutants from adipocytes and increased their levels in the circulation (45). It was reported that the increase of toxic chemicals in serum is proportional to the weight loss (45). A weight loss of 10% was related to an increase of persistent organic pollutants up to 20% in blood over 4–6 months (46, 47). In this study, a weight loss by 3.6% was reached after 10 fasting days and by 10.3% after 4 weeks.

We did not document an enhanced excretion of heavy metals or of glyphosate in urine. The excretion of heavy metals in urine may have been different during the first days of the treatment. This will have to be investigated. By contrast, urinary levels for these compounds were decreased in some cases. It is possible that the decreased dietary intake could have masked the elimination. The high level of water daily ingested may have also contributed to heavy metal elimination since significant proportion of arsenic can be excreted in urine after ingestion (48). Clinical pharmacokinetics includes four components, namely the absorption, distribution, metabolism and excretion (ADME). Our study only evaluated excretion and further studies would be needed to achieve a comprehensive evaluation of chemical pollutant pharmacokinetics during fasting. In comparison with 102 Germans the load of heavy metals in urine seemed to reflect a healthy study population (36). The determination of heavy metal burden directly measured by adipose tissues biopsy could be a suitable way to further investigate changes in bioaccumulation. Our study consisted of a snapshot of urinary pollutants excretion after 10 fasting days. Another option could be to measure pharmacokinetics at different time points to identify the kinetics of elimination. Altogether, little is known about the kinetics of accumulation and washout of these compounds from hair or adipose tissues.

Long-term fasting caused a substantial weight loss in all subjects. On the one hand, weight loss can increase blood levels of bioaccumulated pollutants which are released into the bloodstream as the adipose tissue is used as a source of energy (49). On the other hand, heavy metal body burden can contribute to variations in human weight loss. Body fat percentage correlated with blood levels of lead, cadmium and mercury in a Korean Adult Population (50). Body burdens of lead, cadmium, cobalt, and cesium negatively associated with obesity, while positive association were detected with barium and thallium, in the National Health and Nutrition Examination Survey (NHANES) from 1999–2000 to 2001–2002 (51, 52). A possible explanation is that heavy metals generated metabolic and endocrine disruptions (53). This is also suggested in our study by the positive correlation between arsenic levels and the BMI of the patients. Although it is unclear whether fasting would cause an acceleration of xenobiotics metabolism, leading to a detoxification, our previous studies showed that fasting can enhance protection against cellular damages caused by ROS production (32, 33). In the present cohort, we demonstrated that the total antioxidant capacity significantly increased after 10 fasting days (32, 33).

The decrease in the rate of oxidative damages, and the increase potential for protection against these damages, may explain why we observed that symptoms like fatigue, sleep disorder, headache and hunger, decreased during long-term fasting, indicating that the subjects felt well. The occurrence and intensity of nausea and back pain were at very low levels. As previously described, the well-being of the participants, together with the acceptance and compliance to the protocol, are key elements for a successful fasting procedure (27, 29). Life style changes are also recommended. Studies have demonstrated that long-term fasting is often accompanied by a change toward a healthier life style including an improved eating behavior and an increased physical activity (54). In this study, we also observed changes in life style habits like the enhanced organic food consumption. In general, organic food consumers tend to have a higher awareness toward a healthier life style which explains why large epidemiological studies often found that the consumption of an organic diet correlates with beneficial health effects (55). The lower intake of food contaminated with residues of pesticides including also heavy metals or antibiotics could participate to this health effect although a direct causal link has not been demonstrated. The consumption of organic food further comprises a predominantly plant based nutrition with a conscious selection of dairy products and meet from organic husbandry (55). The described changes in nutritional habits could contribute to the observed maintenance of the reduced BMI 4 weeks after fasting. The diminished contamination after fasting due to life style changes could contribute to lower self-reported body discomforts. However, the establishment of a causative link between the decreased exposure to pollutants and the reduction of symptoms of chronic body discomfort could only be hypothesized, and would need further investigation.

Urinary glyphosate was found in only 8.3% of the subjects before fasting. This low detection frequency in comparison to other assessments (56, 57) may be explained by the higher specificity of the mass spectrometry used in this study (37) or the general healthy life style of the subjects. Some other published analyses have used immunodetections (i.e., ELISA) which could have led to more artifacts by cross reactions with other compounds since the most commonly used ELISA assay for glyphosate has never been fully validated for human urine. It is also possible that this cohort of health-conscious subjects and their general healthy life style makes them less exposed to glyphosate.

Hair samples can provide more long-term information about the exposure to toxic compounds (58). Reference values for metal content in hair have been reported for healthy individuals in Supplementary Table 1 (59). These reference levels were comparable to the metal concentrations found in the present study group. Arsenic, cobalt, and nickel were mostly below the detection limit in hair samples in this study, which suggests that these elements are rapidly from the body through urine (3). Hair levels of mercury and chromium were unchanged, whereas lead levels significantly decreased. The decrease in lead concentrations may reflect the lower dietary exposure as food intake was interrupted during fasting. In another study, a calorie reduced diet for 4 weeks in 15 subjects led to a significant decrease of arsenic in hair (23).

Limitations of the study include the high amount of non-detectable values, pointing to the need of more sensitive technologies and a higher number of participants. In addition, a closer monitoring of the kinetics could bring further insides into the excretion of heavy metals and glyphosate, or other pesticides. Nevertheless, this “before–after” analysis lays a good foundation for further studies, which should compare levels of xenobiotics in different compartments like urine, blood, and fat tissue. This will contribute to a better understanding of the exchanges between these compartments during long-term fasting. Moreover, the explorative hair analysis presented in this study should be extended to a higher number of subjects with different time points of sampling. Future studies should also assess more long-term changes in exposure to heavy metals and pesticides, or other pollutants such as plasticisers, brominated and fluorinated compounds. The evaluation of the change in their bioavailability caused by fat mobilization during fasting can help understanding their impact on health.

Altogether, studies about the effects of fasting on the elimination of environmental pollutants are scarce. Our study showed a reduction in the urinary levels of arsenic and nickel, as well as a reduction in hair lead levels during a 10-days fasting period. In parallel, symptoms like fatigue, sleep disorder, headache and hunger were diminished, reflecting the tolerability and benefit of this procedure. Body discomfort symptoms were diminished 4 weeks after food reintroduction. More studies are needed to understand how therapeutic intervention can influence the detoxification capacity of the body for pollutants.

## Data Availability Statement

The raw data supporting the conclusions of this article will be made available by the authors, without undue reservation.

## Ethics Statement

The study involving human participants was reviewed and approved by Landesärztekammer Baden-Württemberg Ethik-Kommission Liebknechtstr. 33 70565 Stuttgart.

## Author Contributions

G-ÉS had the idea for this study. G-ÉS, FWT, and FG conceived and conceptualized the study. FG was project manager, coordinated study conduction, data collection and drafted the manuscript. RM performed the bioinformatics and statistical analysis. VP performed parts of the laboratory analyses (hair measurements). G-ÉS and RM contributed to data interpretation and the writing of the manuscript. All authors approved the final manuscript.

## Funding

The study was financed by Amplius GmbH, Überlingen, Germany. This company was in charge of the research of and funded by Buchinger Wilhelmi.

## Conflict of Interest

FWT and FG are employees at Buchinger Wilhelmi GmbH. RM is consultant of Amplius GmbH and receive financial compensation for this role. RM has served as a consultant on glyphosate risk assessment issues as part of litigation in the US over glyphosate health effects. This study received funding from Amplius GmbH, Überlingen. The funder had the following involvement with the study: it coordinates the management of the studies for Buchinger Wilhelmi. The remaining authors declare that the research was conducted in the absence of any commercial or financial relationships that could be construed as a potential conflict of interest.

## Publisher's Note

All claims expressed in this article are solely those of the authors and do not necessarily represent those of their affiliated organizations, or those of the publisher, the editors and the reviewers. Any product that may be evaluated in this article, or claim that may be made by its manufacturer, is not guaranteed or endorsed by the publisher.
