# Supplementary material for: Excretion of Heavy Metals and Glyphosate in Urine and Hair Before and After Long-Term Fasting in Humans
Source: Front Nutr. 2021 Sep 28;8:708069. doi: 10.3389/fnut.2021.708069 (PMC8505741; doi:10.3389/fnut.2021.708069)
Supplement: Supplementary file 1 [file Table_1.DOCX]

Supplementary Material

**Supplementary Table 1. Reference values of heavy metals in urine and hair.**

|  | **Urine** | | | **Hair** | | |
| --- | --- | --- | --- | --- | --- | --- |
|  | Heitland and Köster 2021 (n=102) | | | Goullé et al. 2005 (n=45) | | |
| **Metabolite** | **Mean (µg/l)** | **Range** | **5^th^-95^th^ percentile** | **Median (ng/mg)** | **Range** | **5^th^-95^th^ percentile** |
| Arsenic | 10.3 | 0.5-71 | 1.1-44 | 0.05 | - | 0.03-0.08 |
| Chromium | 0.17 | <0.1-1.1 | <0.1-0.35 | 0.20 | - | 0.11-0.52 |
| Cobalt | 0.34 | 0.026-2.6 | 0.039-1.3 | 0.023 | - | 0.004-0.14 |
| Lead | 0.46 | 0.06-2.7 | 0.095-1.0 | 0.41 | - | 0.13-4.57 |
| Mercury | 0.25 | <0.02-0.78 | 0.1-0.63 | 0.66 | - | 0.31-1.66 |
| Nickel | 1.35 | <0.4-6.2 | <0.4-4.0 | 0.23 | - | 0.08-0.90 |
